# Consultations with locum doctors in UK general practice: longitudinal analysis of electronic health records

**DOI:** 10.3399/BJGP.2025.0298

**Published:** 2026-02-10

**Authors:** Thomas Allen, Christos Grigoroglou, Kieran Walshe, Gemma Stringer, Jane Ferguson, Evangelos Kontopantelis, Stuart Stewart, Charlotte Morris, Darren M Ashcroft

**Affiliations:** 1 Manchester Centre for Health Economics, Division of Population Health, Health Services Research and Primary Care, University of Manchester, Manchester, UK; 2 Danish Centre for Health Economics, University of Southern Denmark, Odense, Denmark; 3 Alliance Manchester Business School, University of Manchester, Manchester, UK; 4 Health Services Management Centre, School of Social Policy and Society, University of Birmingham, Birmingham, UK; 5 NIHR School for Primary Care Research, Centre for Primary Care, Division of Population Health, Health Services Research and Primary Care, University of Manchester, Manchester, UK; 6 Division of Informatics, Imaging and Data Sciences, University of Manchester, Manchester, UK; 7 Division of Family Medicine, Yong Loo Lin School of Medicine, National University of Singapore, Singapore, Singapore; 8 Donal O’Donoghue Renal Research Centre, Northern Care Alliance NHS Foundation Trust, Salford, UK; 9 Centre for Pharmacoepidemiology and Drug Safety, School of Health Sciences, Faculty of Biology, Medicine and Health, University of Manchester, Manchester, UK; 10 NIHR Greater Manchester Patient Safety Research Collaboration, University of Manchester, Manchester, UK

**Keywords:** delivery of health care, general practice, locums, primary health care, retrospective study, workforce

## Abstract

**Background:**

Locum doctors are vital for maintaining healthcare provision, especially in general practice. However, their levels of use, role, and impact remain relatively understudied and official statistics cannot be compared between UK countries.

**Aim:**

To explore locum use more comprehensively in the UK, over time and across geographies.

**Design and setting:**

A retrospective cohort study using the Clinical Practice Research Datalink (CPRD) GOLD from April 2010 to March 2022, analysing consultations across UK general practices.

**Method:**

Consultation types were categorised by GP type (locum versus permanent). Multilevel mixed-effects logistic regressions modelled the association between locum consultations and patient and practice characteristics.

**Results:**

Between 2010 and 2022, 914 UK general practices contributed to the dataset. UK locums provided more care than previously estimated, with a 2019 (pre-COVID-19 pandemic) mean of 14.9% (standard deviation 20.7) and median of 7.5% (interquartile range 2.3–17.5) of all consultations, indicating substantial variation. Over time, the use of locums in the UK remained relatively stable, but this masks different trends within the UK. The study found that use in England is increasing, in Scotland and Northern Ireland decreasing, and in Wales is flat. Regression analysis found that variation in locum use was largely unexplained by patient and practice characteristics.

**Conclusion:**

This study indicates higher use of locums across the UK than available NHS statistics would suggest. These differences are probably because of alternative ways of measuring GP activity (consultations versus hours worked). Regional and country differences highlight diverse workforce challenges where local solutions may be needed. These findings contribute to understanding NHS workforce dynamics and may help inform strategies for primary care service delivery.

## How this fits in

Measuring locum contribution in the UK is difficult, as official statistics may underestimate direct care and are not comparable across countries. Using consultation rates for locums and permanent GPs provides a stable, comparable measure over time. Locum contribution is higher than previously estimated and rising in England, but falling in Scotland and Northern Ireland, and stable in Wales. These findings shed light on NHS workforce dynamics and may inform primary care planning.

## Introduction

In most developed healthcare systems, including the NHS, primary care plays a central role in delivering healthcare services. It encompasses a wide range of essential functions such as initial health consultations, diagnosing and managing diseases, and promoting disease prevention through screening and health promotion activities. Primary care acts as the first point of contact for individuals seeking healthcare services and is responsible for providing comprehensive, accessible care; as such, access to primary care represents a fundamental aspect of a high-quality healthcare system.^1,2^

Better availability of primary care services has demonstrated associations with reduced premature mortality,^3^ all-cause mortality, decreased hospital admission rates, and lower healthcare expenditures.^4^ Nevertheless, there are currently several challenges facing general practice in reducing barriers and inequalities in access.^5,6^ The UK faces insufficient personnel and resources to address the escalating demands and complexities of patients, which directly has an impact on patient satisfaction, the quality of patient care, and the experience of healthcare staff.^7,8^ Employing a flexible GP workforce may help relieve some of these pressures relating to access, although the use of locums for patients with greater need has been questioned.^9^ Overall, limited research exists on the use of temporary GPs, commonly referred to as locums, in the UK.^10^

In 2019, it was reported that locums accounted for 3.3% of the total GP workforce, using official NHS data and full-time equivalent (FTE) figures,^11^ and the most recent official NHS data report similarly low values.^12^ However, this seemingly low value is at odds with some other reports suggesting up to 39% of GPs worked as locums in 2016.^13^ These apparently contradictory results may suggest that, although many GPs do some locum work alongside their normal workload, fewer work wholly as locums and so locums provide a small share of the primary care workforce. In addition, it is known that GPs only spend around 60% of their time on direct patient care^14^ and this figure may be different for locum GPs.^15^ Thus, the proportion of patient care provided by locums cannot be determined from workforce headcounts, and using such counts may underestimate the care provided by locums. It seems likely that different ways of measuring locum work will lead to different estimates and conclusions.

Having a clearer understanding of the proportion of direct patient care provided by locums, how this has changed over time, and how this varies by practice and geography have important policy implications. Policy relevance becomes evident if the contributions locums make to patient care are underestimated in official statistics based on headcounts. Furthermore, a better understanding of the level of locum care will help contextualise some of the differences between locums and permanent GPs that have been reported in the literature, such as prescribing and referral behaviour; quality, safety, and working conditions;^16,17^ and continuity of care.^9,18^

These issues are addressed by investigating the proportion of patient care provided by locums using a large general practice database of electronic health records. The primary aim was to quantify and compare general practice locum use using consultation rates as an alternative to FTE data. The study also aimed to investigate patient and practice characteristics that are associated with locum consultations, and to describe the variation in locum use over time and by geography.

## Method

### Data

Clinical Practice Research Datalink (CPRD) GOLD is a large database of anonymised primary care medical records. It contains complete electronic health records for >14 million patients in general practices using the Vision system, with the healthcare events (diagnoses, treatments, referrals, tests, and prescriptions) recorded using coding systems.^19^ The database included practices from England, Wales, Scotland, and Northern Ireland, and is broadly representative of the UK population in terms of age, sex, and ethnicity, although larger practices are over-represented. The data have been shown in numerous validation studies to be generally of high quality.^20,21^ For the analysis, all UK practices within CPRD GOLD that met prespecified data-entry quality criteria that were considered to be up to research standard were included.^22^

### Analyses

A retrospective cohort study was conducted of GP consultations at 914 general practices in the UK from 1 April 2010 to 31 March 2022. Consultation information was aggregated within each financial year (which runs from 1 April to 31 March) for each active patient registered for at least 1 day during the respective year. Patients who had a recorded year of death before the beginning of the period of study were excluded from the analyses.

Clinical consultations were identified and grouped based on the variable ‘consultation type’ that were completed by any GPs, identified by the ‘staff role’ variable (See Supplementary Tables S1 and S2 for codes). Consultations were grouped into three types:

- all consultations;
- face-to-face consultations; and
- telephone and online consultations.

These groups were further split by whether the consultation was with a permanent GP or a locum. Consultations were also grouped by day of the week to determine if locum use varied between weekday and weekend. Owing to the very low number of emergency, out-of-hours, and home visit consultations these were not examined separately.

#### UK analysis

To understand time trends and variation in the proportion of consultations performed by locums in the UK, descriptive and graphical analysis of locum consultation rates were performed. This was done for the 914 UK practices, and also separating those in England, Wales, Scotland, and Northern Ireland.

To quantify the association between GP locum consultations and patient and practice characteristics, multilevel mixed-effects logistic regression models were used. Analyses were conducted at the patient level, and accounted for the nested structure of patients within general practices (random intercept for practice). To do this, the descriptive analysis sample was restricted by randomly selecting one consultation event for each patient within each financial year and aligning all the covariates to that specific event date for the patient. This approach gave equal weights to patients, and therefore limits the potential confounding introduced by patients with very poor health who are likely to visit numerous times within a year. To determine if the study’s findings were robust to this sample restriction, regression analysis was also performed on a random sample consisting of 20% of all patients, and all consultation events were extracted for these patients.

The outcome was a binary variable indicating whether the consultation was delivered by a GP locum rather than a permanent GP. Model covariates were: patient sex, age, and the Cambridge multimorbidity score.^23^ To estimate time trends in the locum consultation rate, a measure of linear time in years was interacted with variables indicating the practice location (England, Wales, Scotland, and Northern Ireland), yielding the time trend for locum use in each country. There were no missing data. Stata (version 17) was used for the principal data cleaning, management, and analyses.

#### England analysis

As additional data linkages were available only for practices in England, additional analysis were performed for these practices and included area-level deprivation as measured by the Index of Multiple Deprivation (IMD) 2015 quintile;^24^ rural/urban area of patients’ place of residence; practice list size; geographical region; and year. The model for English practices was estimated with the same multilevel mixed-effects logistic regression used for UK practices. Categorical variables for each year allowed these models to consider nonlinear effects of time. Fewer practices from England contribute to CPRD GOLD over time, particularly after the COVID-19 pandemic, driven by the changing landscape of clinical system use.^25^ Therefore, these additional analyses were performed for all English practices (*n* = 407) and also for practices contributing in every year before 2020 (*n* = 85).

## Results

### Descriptive analysis

Table 1 shows that the number of practices across all UK countries that participated in CPRD GOLD varied from 870 in 2010 to 422 in 2021. This reduction is mostly because of English practices moving away from the Vision IT system.^25^ In total, data were available for 914 general practices in the UK: 511 practices from England, 234 practices from Scotland, 127 from Wales, and 42 from Northern Ireland. Of these, 381 practices contributed data in every year. The geographical distribution of practices across all UK countries, and regions of England, is shown in Supplementary Table S3.

**Table 1.** Consultations by locums and permanent GPs by consultation type in the UK, April 2010 to March 2022^a^

Financial year	All consultations, *n*		Face-to-face consultations, *n*		Telephone and online consultations, *n*		Total patients, *n*	Practices, *n*
	All GPs	Locums	All GPs	Locums	All GPs	Locums		
2010	20 505 515	2 160 110	18 732 124	2 031 389	1 773 391	128 721	4 184 690	870
2011	20 747 849	2 292 973	18 848 827	2 148 948	1 899 022	144 025	4 241 700	876
2012	21 183 340	2 369 466	19 080 750	2 201 442	2 102 590	168 024	4 245 588	854
2013	20 655 419	2 241 487	18 436 232	2 061 195	2 219 187	180 292	4 127 214	836
2014	19 140 726	2 154 532	16 964 052	1 969 834	2 176 674	184 698	3 836 633	789
2015	16 582 566	1 917 867	14 682 494	1 757 568	1 900 072	160 299	3 392 982	716
2016	14 376 977	1 665 881	12 769 293	1 546 654	1 607 684	119 227	2 895 826	621
2017	12 801 485	1 482 861	11 391 487	1 377 101	1 409 998	105 760	2 603 629	559
2018	11 892 772	1 407 472	10 619 499	1 310 867	1 273 273	96 605	2 443 610	524
2019	10 987 908	1 311 290	9 719 650	1 214 468	1 268 258	96 822	2 290 261	500
2020	8 935 244	1 003 237	5 910 979	676 680	3 024 265	326 557	1 789 703	461
2021	6 499 969	756 361	4 421 649	527 265	2 078 320	230 096	1 551 491	422

^a^Figures calculated over financial years, that is, 2010 refers to April 2010 to March 2011.

For the descriptive analysis, the cohort consisted of all 8 133 324 unique patients, with 184 309 770 recorded consultations. Totals for the different types of consultations by type of GP and over time are shown in Table 1. For example, in 2019 there were almost 11 million GP consultations and 1.3 million of these were by a locum GP, giving a locum consultation proportion of 11.8%. Telephone and online consultations remained relatively low and stable until 2020, when they increased during the COVID-19 pandemic.

In Table 2, statistics are presented on the percentage of all GP consultations that were performed by locums, calculated at the practice level. The mean locum consultation percentage was 12.8% (standard deviation [SD] 17.7) in 2010 and rose to 14.9% (SD 20.7) in 2019. The 50th percentile was lower than the mean at 7.5% in 2019, which, along with the large SD, suggests significant variation in the use of locums at a practice level. The 75th percentile reveals that a quarter of practices were using locums for >17.5% of consultations in 2019.

**Table 2.** Locum consultations as a percentage of GP consultations, UK April 2010 to March 2022^a^

Category	Year, locum consultations as % of GP consultations											
	**2010**	**2011**	**2012**	**2013**	**2014**	**2015**	**2016**	**2017**	**2018**	**2019**	**2020**	**2021**
Mean	12.8	13.5	13.7	13.4	13.3	13.8	14.2	14.3	15.0	14.9	14.1	14.8
Standard deviation	17.7	18.4	18.7	18.6	18.2	18.7	19.4	19.4	20.6	20.7	20.3	20.4
25th percentile	1.1	1.2	1.3	0.9	1.2	1.4	1.2	1.6	2.0	2.3	2.1	1.7
50th percentile	6.0	6.3	6.7	6.0	7.0	7.2	6.9	7.0	7.3	7.5	6.4	7.4
75th percentile	16.8	18.1	17.9	16.4	17.1	17.3	18.5	18.2	18.7	17.5	16.4	18.5

^a^Figures calculated at a practice level and over financial years, that is, 2010 refers to April 2010 to March 2011.

**Figure 1.**
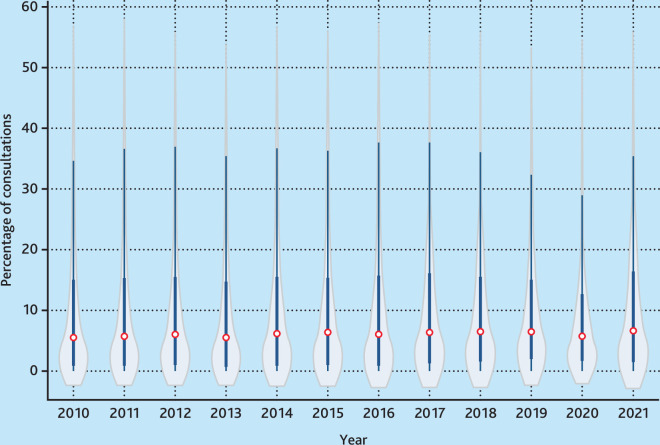
Locum consultations as a percentage of all GP consultations in the UK, April 2010 to March 2022. Violin plots can be interpreted as box plots (including the median as a marker and a box indicating the interquartile range), overlaid with the density of the distribution for enhanced visualisation. Calculated over financial years, that is, 2010 refers to April 2010 to March 2011.

Violin plots showing the percentage of locum consultations from April 2010 to March 2022 are presented in Figure 1. Although there were modest increases in locum use over time, the variation between practices was high in all years, with some practices using two or three times the median value. The equivalent plots for face-to-face and telephone and online consultations are provided in Supplementary Figures S1 and S2 and show similar patterns.

Regional variation in locum consultations, aggregated over the period April 2010 to March 2022, for all UK countries and English regions is depicted in Supplementary Figure S3, including variation between and within areas.

**Figure 2.**
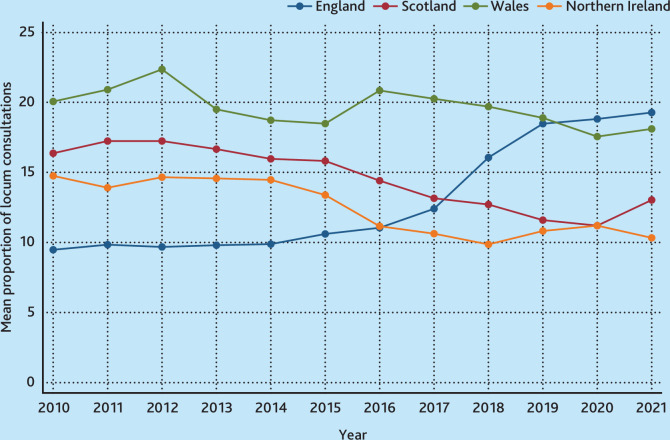
Mean percentage of locum consultations by country, April 2010 to March 2022. Each dot represents the mean percentage of locum consultations by country and year; connected dots visualise the time trend.

Figure 1 masks changes over time that differ between England, Wales, Scotland, and Northern Ireland; these time trends are depicted in Figure 2. In all years except 2020 and 2021, practices in Wales had the highest use of locums, approximately 20.0% of consultations. Practices in Scotland and Northern Ireland had similar levels that gradually reduced over time. Practices in England had relatively low use of locums in 2010 (10.0% compared with 20.0% for Wales), and this increased from 2014 to a high in 2021 of approximately 19.0%. Supplementary Figure S4 presents the equivalent for a balanced panel of practices contributing in all years and the time trends are similar.

Supplementary Figures S5–S9 show the mean proportion of locum consultations by day of the week in 2018/2019. Across the whole of the UK, locum GPs provided on average 15.0% of total care with minimal variation between weekdays or the weekend, although the volume of consultations is lower at the weekend. In England, locums provided a higher proportion of consultations at the weekend, in Scotland and Wales the proportion was lower, and in Northern Ireland the weekend proportion was similar to the weekday proportion.

### UK regression analysis

After randomly selecting a consultation for each patient in each year, the sample was 37 543 165 consultations across 913 practices in the UK between April 2010 and March 2022. The results from the multilevel fixed-effects model are reported in Table 3.

**Table 3.** Mixed-effects logistic regression for locum consultations over time in the UK^a^

Variable	Odds ratio	95% CI	*P*-value
**Female sex**	0.990	0.980 to 0.992	<0.001
**Age**	0.996	0.996 to 0.996	<0.001
**Cambridge multimorbidity score**	0.954	0.952 to 0.955	<0.001
**Country yearly time trends**			
England	1.064	1.064 to 1.065	<0.001
Wales	1.004	1.003 to 1.004	<0.001
Scotland	0.947	0.946 to 0.948	<0.001
Northern Ireland	0.956	0.955 to 0.958	<0.001
**Constant**	0.083	0.073 to 0.094	<0.001

^a^Estimated using multilevel mixed-effects logistic regression models, patients nested within practices. Sample consultations, *n* = 37 543 165; sample practices, *n* = 913.

The effect sizes for patient characteristics were generally small, for example, the odds a female patient would have a consultation with a locum were 1.0% lower. Each year a patient’s age was associated with a decrease in odds of locum consultation of 0.4%. Patients with a higher comorbidity score were 4.6% less likely to have a consultation with a locum. Yearly trends show that practices in England increased their locum consultation rate by 6.4%, on average, between 2010 and 2021. For Wales, the trend is essentially flat during the period. Scotland and Northern Ireland both decrease their use of locums over this period by 5.3% and 4.4%, respectively (Table 3). Models including practice list size would not converge, but results from the English practice sample suggest that list size has only a small association with locum use.

As a measure of model performance, the receiver operating characteristic area under the curve value of 0.74 suggested acceptable, but not exceptional, discriminative power. Based on the characteristics included, the model assigns a higher predicted probability to patients who actually saw a locum 74.0% of the time.

Regression results using all consultations for a random sample of 20.0% of patients find very similar results and are shown in Supplementary Table S4.

### English regression analysis

The sample from England consisted of 13 696 455 consultations across 407 practices between April 2010 and March 2022. The results from the multilevel fixed-effects model are reported in Table 4. The effect sizes for patient characteristics were similarly small, as in the UK models.

**Table 4.** Mixed-effects logistic regression for locum consultations over time in England^a^

Variable	Odds ratio	95% CI	*P*-value
**Female sex**	1.012	1.008 to 1.016	<0.001
**Age**	0.994	0.994 to 0.994	<0.001
**Cambridge multimorbidity score**	0.931	0.928 to 0.933	<0.001
**Patient list size**	1.000	1.000 to 1.001	<0.001
**IMD quintile**			
1 (most deprived)	Reference		
2	1.013	1.007 to 1.020	<0.001
3	1.014	1.007 to 1.021	<0.001
4	1.019	1.012 to 1.027	<0.001
5 (least deprived)	1.020	1.012 to 1.028	<0.001
**Rural practice**	1.003	0.993 to 1.012	<0.537
**Region**			
Midlands	Reference		
North East	0.158	0.039 to 0.641	<0.010
North West	1.308	0.654 to 2.614	<0.448
Yorkshire and Humber	0.773	0.236 to 2.532	<0.670
East of England	0.399	0.177 to 0.901	<0.027
London	2.059	1.039 to 4.078	<0.038
South East	1.091	0.570 to 2.088	<0.792
South West	1.648	0.781 to 3.481	<0.190
**Year**			
2010	Reference		
2011	1.075	1.067 to 1.083	<0.001
2012	1.082	1.074 to 1.090	<0.001
2013	1.093	1.085 to 1.102	<0.001
2014	1.212	1.203 to 1.222	<0.001
2015	1.237	1.227 to 1.248	<0.001
2016	1.322	1.310 to 1.334	<0.001
2017	1.620	1.604 to 1.636	<0.001
2018	1.756	1.738 to 1.774	<0.001
2019	1.965	1.944 to 1.987	<0.001
2020	2.024	1.995 to 2.053	<0.001
2021	2.617	2.571 to 2.663	<0.001
**Constant**	0.045	0.027 to 0.075	<0.001

^a^Estimated using multilevel mixed-effects logistic regression models, patients nested within practices. Sample consultations, *n* = 13 696 455; sample practices, *n* = 407. IMD = Index of Multiple Deprivation.

Patient list size had a small association with the rate of locum consultations; an increase of 1000 patients was associated with increased odds of 1.6%. In terms of deprivation, variation between deprivation quintiles was very small: the largest difference observed was a 2% increase in the odds of locum consultation between the most and the least deprived quintiles (Table 4).

Variation was more pronounced between regions, although not always statistically significant. The largest differences observed here were the odds of a locum consultation being double in London and approximately 84.0% lower in the North East, when compared with the Midlands, which was this study’s reference group. As seen in the UK models, over the study period, later years were associated with more locum consultations. For example, in 2021 the odds a patient had a consultation with a locum were 2.617 (95% confidence interval = 2.571 to 2.663) times greater compared with 2010 (Table 4).

### Pre-pandemic regression analyses

Additional analysis repeated the regression shown in Table 4, restricting the sample to the pre-pandemic years from April 2010 to March 2020 and including only the 85 practices from England that appeared in all of those years (Supplementary Table S5). The results were very similar and the increase in locum use, of approximately two times from 2010 to 2020, remained.

## Discussion

### Summary

This study provides valuable evidence on the scale and scope of locum use in UK primary care over a long time period between April 2010 and March 2022. The findings suggest that locum GPs provided around 10.0% of GP consultations (2015 median 7.2% and mean 13.8%) but with considerable variation between practices, between UK countries, and over time. For the UK, locum use remained relatively stable over time, but use fluctuates when looking separately at England, Wales, Scotland, and Northern Ireland. Between 2010 and 2022, locum use increased in England, remained flat in Wales, and decreased in Scotland and Northern Ireland. These different trends are seen in the graphical and regression analysis. That patient and practice characteristics only had modest explanatory power, suggests that unobservable characteristics contribute to the variation in locum use. Restrictions on linking CPRD to other data prevents the use of potential explanatory variables such as: regulatory rating; number of full-time GPs or nurses; local workforce policies; opening hours; or GP shortages and turnover rates.

There is also variability in the proportion of care provided by locum GPs across English regions. This suggests that different regions and countries are affected by, and respond to, workforce challenges in different ways. Locums provided very similar proportions of care during weekdays and over the weekend, which highlights the key role played by locums in delivering primary care services throughout the week, and suggests they are not just providing out-of-hours primary care. Overall, the findings provide new insight into the extent of locum use in UK primary care over a longer time period than previous research.

### Strengths and limitations

To the authors’ knowledge, this is the only longitudinal study of locum consultation rates in UK primary care, covering 12 years of data from electronic health records. Other research into the use of locums in primary care has relied on NHS England workforce statistics that measure the numbers of staff (in FTEs or headcount) but not activity in patient care.^11,26^ The reliance on workforce statistics for measuring locum usage also does not allow for controlling for any patient characteristics, as the aggregated data are only published at the practice level. The current work has highlighted the importance of adjusting for such factors and the value of consultations as an alternative measure of locum use. Previous research focused on locum use in England, not in Wales, Scotland, or Northern Ireland, and this current study found different trends of locum use across the four countries of the UK. This research is timely and highly relevant, given workforce challenges faced by primary care in the UK.

However, there are important limitations. First, although the authors of the current study are confident about the reliability of the recorded patient contact data and patient characteristics, less is known about the accuracy of type of consultation, meaning consultations may be recorded as face to face by default. If so, the data could have underestimated the use of telephone and online consultations.

Second, it was not possible to validate the recording of the staff role linked to each staff record, which was then linked to a consultation. This creates a limitation if practice managers were to record locum GPs as permanent GPs, which might be more common with long-term locum GPs and result in underestimating locum use. Relatedly, when changes are made to the staff role (when a permanent GP changes to locum for instance), practice managers have two options: to create a new staff record for the new role or to overwrite the existing record and replace the old role with the new. In the first case, consultations can be separated between roles. In the second case, historic and future consultations will be allocated to the new role. When GPs move practice they are given a new staff record, meaning changes in role involving a new practice are not affected. If the GP stays in the same practice and a new record is created, this causes no limitation. The limitation comes when the GP stays in the same practice but the record is overwritten. Although, as GPs can move from locum to permanent (and vice versa) any bias will not always in the same direction.

Third, CPRD GOLD collects data from practices using the Vision clinical information system and recording activity may differ for practices using other systems, although the authors would not expect that potential variation to affect the findings.

Fourth, CPRD GOLD practices are representative in terms of age, sex, and ethnicity,^19,27^ but contributing practices are not uniformly distributed across the UK and English regions.^25^ Thus, generalisability to every English region could not be confirmed. Related to this is the decreasing number of English practices who contribute data to CPRD GOLD over time and an over-representation of practices from Scotland. This issue was explored in the regression analysis for English practices, first for all 407 practices and then for 85 practices contributing in all years. It is reassuring that these two regressions found similar time trends, suggesting that practices dropping out of CPRD in England does not drive this finding.

### Comparison with existing literature

The unique contribution of this study is the investigation of distribution and trends in the delivery of care by GP locums across UK primary care. This contribution includes being able to compare locum use in England, Scotland, Wales, and Northern Ireland using a common and consistent measure, which is not possible with official statistics owing to changes in methods over time and differences in methods between countries.

In contrast to previous research using NHS Digital data, the current study found that locum use in England is higher and increasing over time.^11^ The authors of the current study suggest three possible explanations for this difference. First, the measure of locum use was different, with CPRD measuring consultations (2015 median 7.2% and mean 13.8%) and NHS Digital measuring FTE hours (mean 3.0%). If locums spend more time providing direct patient care, compared with permanent GPs who may engage more in practice management and training,^15^ some of this difference would be expected. Second, NHS Digital data are based on workforce information submitted by the practice, and concerns have been raised about the reliability and completeness of this information.^11^ Previous qualitative work revealed that practice managers may record long-term locums as permanent members of staff, such as salaried GPs.^28^ Therefore, the figures based on the analyses of NHS Digital data are likely underestimating the proportion of care provided by locum GPs. Finally, it is possible that some of the difference could also be driven by the adjustment for patient characteristics in regression analysis.

Peer-reviewed research on locum use in Scotland, Wales, and Northern Ireland is lacking, but the current findings can be placed in greater context using published official workforce statistics from these countries. However, these comparisons come with a caveat that data collection and reporting differs by country. In Scotland, data from 2019 report 3613 FTEs for all GPs and 273 FTEs for locums, suggesting 7.6% of care is performed by locums.^27^ Contrast this to the finding in the current study for 2019 of 12% of consultations performed by locums in Scotland. In Wales, locum FTEs are not reported until 2022, which complicates comparisons. However, the official statistics report locum FTEs at 5.6% of all GP FTEs.^29^ Contrast this with the finding in the current study for 2021 of 18% of consultations performed by locums in Wales. Northern Ireland do not report locums using FTEs and instead use headcount, reporting that in 2023 there were 1448 GPs and a further 508 locums.^30^

Interestingly, GPs are increasingly expressing concerns about their employment contract,^31,32^ with salaried and locum GPs reporting increasing difficulty finding employment.^33–35^ Such reports are striking in the context of the current study’s findings that show a large contribution of locum GPs to UK primary care over time. Thus, posing the question, if locum GP use is significant and increasing over time, why are salaried and locum GPs now struggling to find work? This may possibly be owing to attempts to curb spending on GP use in primary care in favour of other clinical non-GP roles.^36^ The increasing use of locums in England should also be seen in the wider context of falling numbers of permanent GPs and increasing pressures from growth in demand from patients,^37–40^ both of which may be partly eased with temporary staff.

### Implications for research and practice

The current findings shed light on the level and variation in locum use, and they should be considered alongside the literature comparing locums and permanent GPs with respect to quality, safety, and continuity of care. Although care delivered by locum GPs has not been linked to safety concerns such as hazardous prescribing, it has been linked to higher rates of prescribing for antibiotics and lower rates of tests and referrals, which poses more of a concern if locum use is higher than expected.^41^ Furthermore, locum GPs face substantial challenges, with large variability between practices and areas they work in, more acutely unwell and unfamiliar patients, limited communication, feedback, and support, and professional isolation.^16,17^ These challenges create an environment where quality and safety could be at risk. Patients have different views of locums, often depending on if they have an acute or chronic need, valuing quick access in the former and continuity in the latter.^9^ Patients having continuity with their GP has also been associated with reduced workload for the practice and local hospitals.^18^ If local healthcare needs are to be met, locum GPs need sufficient resources for training and effective integration that would be aligned with the aims set out in the NHS Long Term Plan.^42^

Levels and patterns in the use of locum GPs were observed in the current study that cannot be determined from official statistics alone. The analyses can help with understanding of the NHS workforce and can provide important information useful for planning and delivery of primary care services in the UK. The authors of the current study recommend more accurate recording of locum GP activity across general practices, with workforce information linked across clinical health record systems. This will help support effective workforce planning that is particularly important in the context of current GP employment concerns.

## Data

This study used anonymised patient-level data from the CPRD that are not publicly available owing to confidentiality considerations. However, researchers can access CPRD’s databases by contacting the CPRD. Details of the application process and conditions of access are available at: https://www.cprd.com/dataaccess.
